# Development and validation of a cadaveric porcine pseudotumor model for oral cancer biopsy and resection training

**DOI:** 10.1186/s12909-024-05224-5

**Published:** 2024-03-07

**Authors:** Claire Melchior, Peter Isfort, Till Braunschweig, Max Witjes, Vincent Van den Bosch, Ashkan Rashad, Jan Egger, Matías de la Fuente, Rainer Röhrig, Frank Hölzle, Behrus Puladi

**Affiliations:** 1 Department of Oral and Maxillofacial Surgery, University Hospital RWTH Aachen, Pauwelsstraße 30, 52074 Aachen, Germany; 2 Institute of Medical Informatics, University Hospital RWTH Aachen, Pauwelsstraße 30, 52074 Aachen, Germany; 3 Department of Diagnostic and Interventional Radiology, University Hospital RWTH Aachen, 52074 Aachen, Germany; 4 Institute of Pathology, RWTH Aachen University, 52074 Aachen, Germany; 5 Institute of Pathology, Faculty of Medicine, Ludwig Maximilians University (LMU), 80337 Munich, Germany; 6 Department of Oral and Maxillofacial Surgery, UMCG Groningen, 9713 GZ Groningen, The Netherlands; 7 Cancer Research Center Cologne Essen (CCCE), University Medicine Essen (AöR), 45147 Essen, Germany; 8 Institute of Artificial Intelligence in Medicine, Essen University Hospital, 45131 Essen, Germany; 9 Chair of Medical Engineering, RWTH Aachen University, 52074 Aachen, Germany

**Keywords:** Simulation training, Models, anatomical, Margins of excision, Mouth neoplasms, Biopsy, Surgical oncology, Education, dental, Oral surgical procedures

## Abstract

**Objective:**

The gold standard of oral cancer (OC) treatment is diagnostic confirmation by biopsy followed by surgical treatment. However, studies have shown that dentists have difficulty performing biopsies, dental students lack knowledge about OC, and surgeons do not always maintain a safe margin during tumor resection. To address this, biopsies and resections could be trained under realistic conditions outside the patient. The aim of this study was to develop and to validate a porcine pseudotumor model of the tongue.

**Methods:**

An interdisciplinary team reflecting various specialties involved in the oncological treatment of head and neck oncology developed a porcine pseudotumor model of the tongue in which biopsies and resections can be practiced. The refined model was validated in a final trial of 10 participants who each resected four pseudotumors on a tongue, resulting in a total of 40 resected pseudotumors. The participants (7 residents and 3 specialists) had an experience in OC treatment ranging from 0.5 to 27 years. Resection margins (minimum and maximum) were assessed macroscopically and compared beside self-assessed margins and resection time between residents and specialists. Furthermore, the model was evaluated using Likert-type questions on haptic and radiological fidelity, its usefulness as a training model, as well as its imageability using CT and ultrasound.

**Results:**

The model haptically resembles OC (3.0 ± 0.5; 4-point Likert scale), can be visualized with medical imaging and macroscopically evaluated immediately after resection providing feedback. Although, participants (3.2 ± 0.4) tended to agree that they had resected the pseudotumor with an ideal safety margin (10 mm), the mean minimum resection margin was insufficient at 4.2 ± 1.2 mm (mean ± SD), comparable to reported margins in literature. Simultaneously, a maximum resection margin of 18.4 ± 6.1 mm was measured, indicating partial over-resection. Although specialists were faster at resection (*p* < 0.001), this had no effect on margins (*p* = 0.114). Overall, the model was well received by the participants, and they could see it being implemented in training (3.7 ± 0.5).

**Conclusion:**

The model, which is cost-effective, cryopreservable, and provides a risk-free training environment, is ideal for training in OC biopsy and resection and could be incorporated into dental, medical, or oncologic surgery curricula. Future studies should evaluate the long-term training effects using this model and its potential impact on improving patient outcomes.

## Introduction

The worldwide incidence of oral cancer (OC) in 2020 was 377,713 new cases, and 177,757 patients with the disease died in the same year, making it one of the most common cancers [1]. Common risk factors for OC are alcohol and tobacco consumption and without treatment will lead to the patient’s death [2]. Although, the overall treatment involves an interdisciplinary team, according to global guidelines surgery is thegold standard for the initial treatment of OC (including tongue cancer). This involves diagnosis by biopsy, followed by usually surgical therapy with resection of the cancer and selective neck dissection to remove the entire tumor and any metastases in the lymph nodes to improve patient outcomes [3].

Paradoxically, OC biopsy or resection is not practiced systematically, either in dental school or in continuing education as a dentist or even as a resident in oral and maxillofacial surgery. In this regard, it has been widely reported worldwide that dental students have a serious lack of knowledge about the diagnosis and treatment of OC [4–9]. This is surprising, as dentists are often the first to take biopsies in the oral cavity in a suspected case [10, 11]. To make matters worse, the majority of dentists report that biopsies are difficult for them [11], while performing of biopsies can lead to several mistakes, such as a too narrow tissue depth, which does not include the epithelium and a few millimeters of the underlying lamina propria, the removal of non-representative tumor segments and crushing artifacts due to inappropriate handling of instruments [12]. However, biopsies are crucial for diagnosis. A delay in diagnosis and treatment initiation can reduce patient outcome [13].

Apart from the requirement of biopsy for diagnosis, one of the primary goals of surgical treatment is surgical resection with adequate resection margins [14, 15], with guidelines suggesting at least 10 mm around the palpable margin of the tumor [3, 16]. The idea behind this is that individual tumor cells may be localized even in areas that appear unchanged macroscopically [17]. This circumstance has been confirmed by 3D morphometric analyses, which have shown that tumor cells appear in the deep invasive front [18]. However, several studies show that specialists do not always achieve the necessary safety margins during tumor resection [15, 17, 19]. Moreover, apart from intraoperative control by the pathologist using frozen sections, initial margins that are too narrow already lead to a worsening of the patient’s outcome [20, 21].

In conclusion, there is room for improvement in the ability of students, dentists, and surgeons to perform accurate biopsies and to achieve sufficient tumor resection margins. Yet, in an RCT, a non-palpable soft tissue tumor phantom model (made of plastic) showed that training medical students supported by surgical navigation feedback led to a reduction in positive margins of tumor resections [22]. Another study of a training program for robotic resection of oropharyngeal cancer improved peripheral resection margins [23]. However, these models are not directly applicable to the circumstances and requirements of OC because the tumors are not palpable and are intended for robotic-assisted surgery, leaving a gap in skills training opportunities. Furthermore, the tongue presents a unique set of challenges: it is highly mobile, flexible, and vascularized while performing many essential functions (tasting, chewing, swallowing, speaking, kissing) and is located in the oral cavity with limited space and visibility for open surgical approaches.

Therefore, establishing a training model for OC and integrating it into curricula could have a positive long-term impact on patient outcomes. It would also address competency-based education, whose key principles are to focus on outcomes, emphasize skills, reduce time-based training, and promote greater learner-centeredness [24]. According to the global oral health competency matrix, basic surgical skills should be taught [25]. In this regard, biopsy as a basic diagnostic tool is a primary part of this. Yet, initial training in the in-patient setting is not advisable, as mistakes could have negative consequences for the patient, including a reduction in survival [20, 21]. However, to the best of our knowledge, there is no model that addresses training for biopsy or resection of OC tumors.

To address this need, an interdisciplinary team developed and validated a porcine tongue pseudotumor. The objectives of this study are: 1.) To develop an off-patient tongue model for practicing biopsies and resections that is easy to prepare, can be visualized by imaging (CT and ultrasound), and allows evaluation of its resection margins in mm, and can be used at all levels of training. 2.) To validate the model with residents and specialists experienced in OC surgery in terms of perceived haptics, radiologic imaging, integration into clinical practice, and usefulness for surgical training.

## Methods

In this development and validation study, an interdisciplinary team from various disciplines (head and neck surgery, dentistry, pathology, radiology, experts in computer-assisted surgery and human biology) developed a pseudotumor model of the porcine tongue.

After the model was developed, a pretrial was conducted with participants. Based on their feedback, a final trial was designed and conducted to validate the model. Inclusion criteria for participants were being a resident or specialist in oral surgery or oral and maxillofacial surgery and giving informed consent to participate. The exclusion criterion was refusal to participate.

The study was approved by the Ethics Committee of RWTH Aachen University Hospital (approval number EK 352/21) and all methods were performed in accordance with the WMA Declaration of Helsinki [26] . The porcine tongues used were purchased from a local butcher as food under the German Food and Feed Act (LFGB) [27] and used and disposed according to the German Animal by-products-Elimination Act (TierNebG) [28].

### Creation of the pseudotumor model

The following requirements were specified for the pseudotumor model: immediate evaluability/feedback, realistic model meaning excisable, not displaceable in surrounding tissue, rounded morphology, palpable, comparable haptics, recognizability on imaging, clearly distinguishable from surrounding tissue (on CT scan and US) and easy storing. Furthermore, the model should be quantitatively and economically feasible, easily integrable into clinical practice, and suitable for all levels of training (palpation, biopsies and resections).

**Fig. 1:**
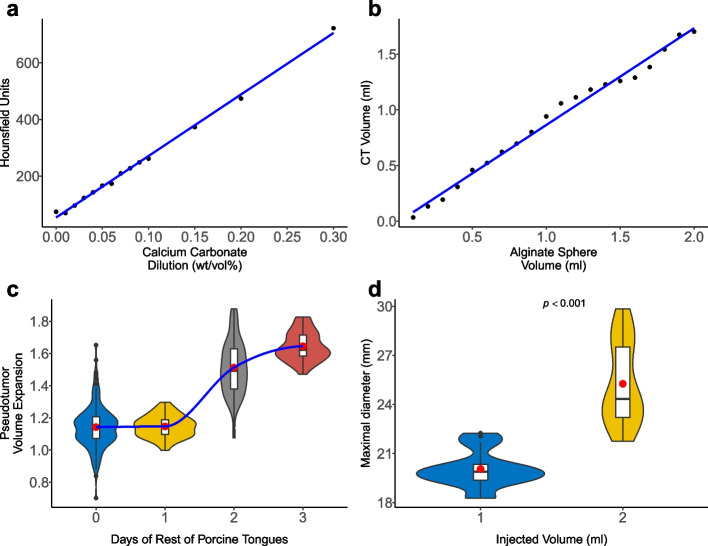
**a** Correlation between calcium carbonate (x-axis) dilution (wt/vol%) and Hus (y-axis) using test specimens measured in a CT scan. **b** Correlation between the volume of freshly prepared alginate spheres (x-axis) and measured volume in CT scan (y-axis). **a**,** b** The dots are the individual measurements. The blue line is the correlation. **c** Volume expansion of the pseudotumor in the porcine tongue in relation to resting days. The blue line shows the LOESS. **d** Maximum tumor diameter on CT scan according to injected volume. **c**,** d** Plots using boxplots and violin plots. Black dots are outliers, and the red dot is the mean. **a-d** All measurements were made with the 3D Slicer in a CT scan

To meet these requirements, the optimal creation processes had to be determined. In this process, more than 400 pseudotumors (Fig. 1b-d) were generated and resected (> 280 by M.C. and B.P. during the development phase; 80 in the pretrial by 10 participants, 40 in the final trial for validation by 10 participants) (Fig. 1).

The final model was produced as follows: 1.5 g sodium alginate powder (CAS#: 9005-38-3) was mixed with 8 ml cold water (≙18.75 wt/vol%). This resulted in a gelatinous mass before curing. To achieve radioopacity in the pseudotumor, we added 2 g of calcium carbonate (CAS#: 471–34-1) per 8 ml of water to the mixture (≙25 wt/vol%). The mixture was then placed into 2 ml syringes, and 1 ml or 2 ml of the mixture was slowly injected into already prepared tissue pockets within porcine tongues using preparatory scissors at four alternating positions (anterior right, posterior right, posterior left, and anterior left). The puncture channel was closed using single-button 4–0 Vicryl sutures (Johnson & Johnson, New Brunswick, New Jersey, US) to prevent the inserted material from accidentally pushing its way out.

**Fig. 2:**
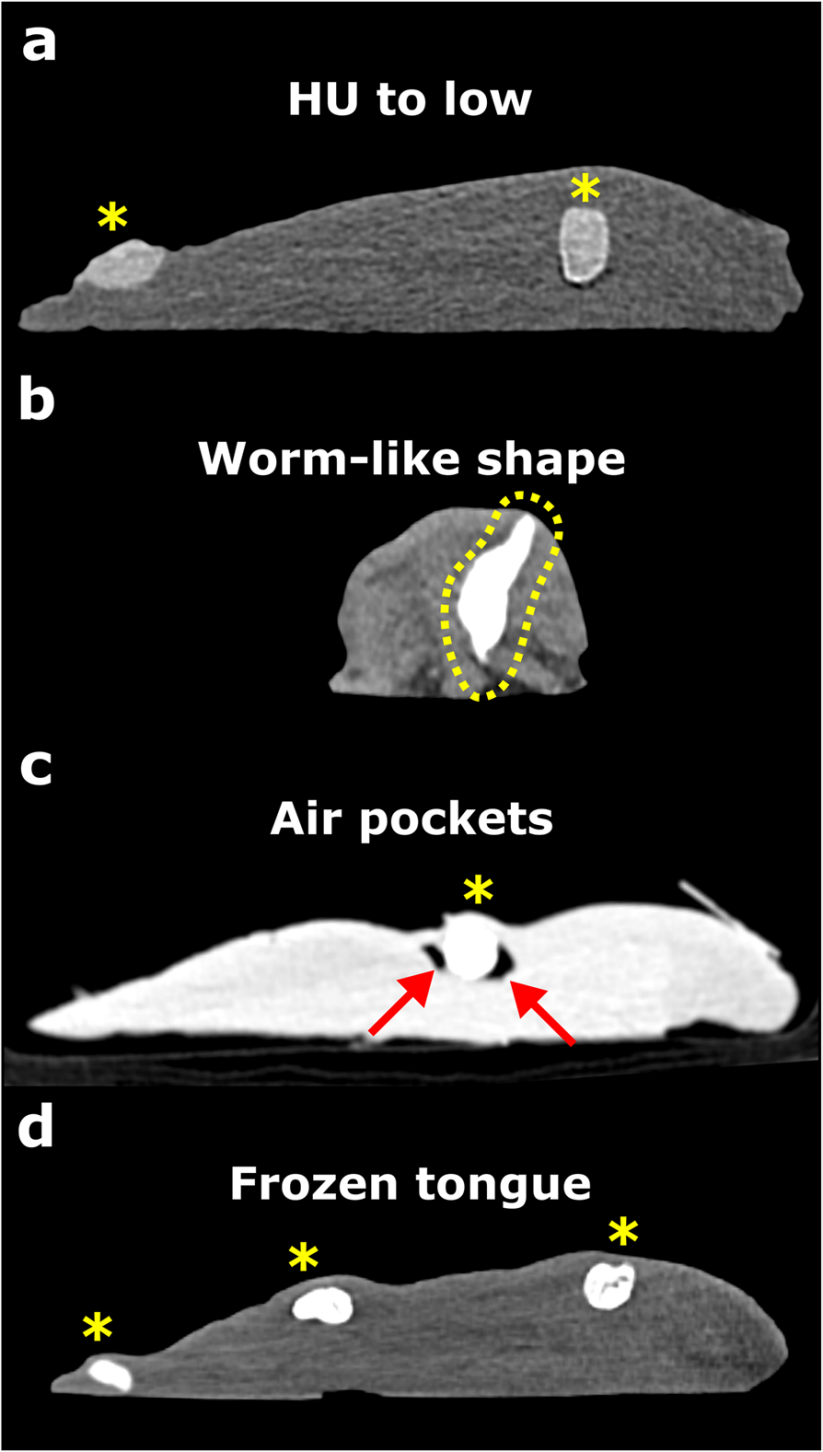
**a** In the CT soft tissue window, a sagittal plane of a porcine tongue with two pseudotumors (yellow *) with a low HU. **b** In the CT soft tissue window, a coronal plane of a porcine tongue with one pseudotumor is shown with a much higher HU and better delimitability, but a worm-like shape (framed in yellow). **c** In the CT lung window, a sagittal plane of a porcine tongue with a pseudotumor showing lack of expansion due to the appearance of air pockets (red arrows) around the pseudotumor due to insufficient rest (< 3 days). **d** Frozen tongue with two pseudotumors. The tissue appears much more homogeneous due to the frozen state

The tongues were then cooled and left to rest for 3 days to allow the mixture to expand to avoid air pockets (Fig. 2c) and to become fixed in the tissue. Computed tomography (CT) scans of the porcine specimens were acquired using a 128-slice dual energy CT scanner (SOMATOM Definition Force, Siemens Healthineers, Forchheim, Germany). CT images were reconstructed in 1 mm slice thicknesses with a 0.7 mm gap by applying a soft tissue kernel (B40). The pseudotumors were delineated well from the tongue tissue as a moderate hyperdense structure.

During the development process, different calcium carbonate concentrations were tested with corresponding pseudotumors that could be visualized radiologically in different ways (Fig. 1a). Initially, we used a lower calcium carbonate concentration, but then decided on a higher concentration, as the pseudotumors are thus more clearly visible on CT, easier to segment, and may not adversely affect the resection margins (Fig. 2a).

For further evaluation, the generated CT datasets were imported into 3D Slicer (version 5.2.2, 10 Jan 2023, www.slicer.org), and the pseudotumors were segmented based on a threshold (150 Hounsfield units [HU]) within the CT dataset. For the pseudotumors with low carbonate wt% (from one of the development phases), we used 50 HU as segmentation threshold (see Fig. 2a). The 50 HU and 150 HU thresholds were chosen based on calcium carbonate concentration using the curve in Fig. 1a.

### Pretrial

A pretrial was conducted to establish the training concept. Ten clinicians in oral and maxillofacial surgery with different surgical experience underwent a training unit with resections of 80 pseudotumors on 20 porcine tongues and haptic evaluations of five porcine tongues. It consisted of two parts: A palpation exercise and a surgical resection training. The participants (all dentists) first had to palpate the five identical tongues and were asked if they could palpate all pseudotumors without knowing how many pseudotumors were in each tongue (one, two, two, three and four pseudotumors). Afterward, they had to resect eight pseudotumors each from two porcine tongues, alternating between using the scalpel and scissors. The selection of the instrument (scalpel or scissors) to resect first was randomized according to a random allocation rule. A Likert questionnaire was filled, which were created considering recommendations [29]. The resections were not analyzed in the pretrial and were discarded. Based on feedback from the pretrial, the study design was modified for the final trial. The Likert scale questionnaires were adapted but not externally validated. Surgical headlights were added to ensure maximum illumination, and CT images were presented during the training session to provide additional guidance during resection. The use of a monopolar was explored, but for safety reasons (risk of severe burns), the use of a monopolar was omitted.

### Final trial

**Fig. 3:**
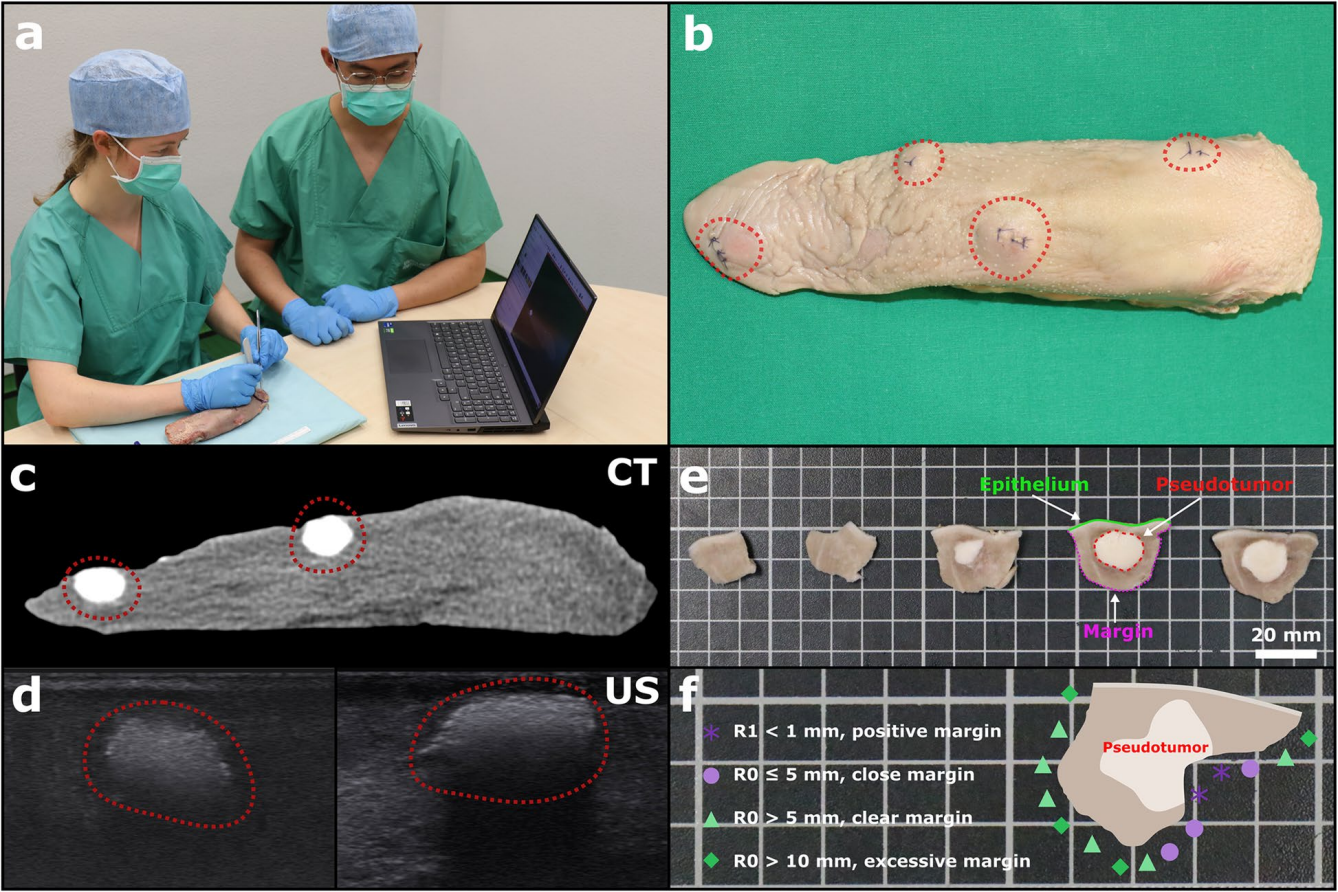
**a** A porcine tongue with pseudotumors lies in front of the participant. The participant (left) holds a scalpel and forceps to perform tumor resection on the pseudotumor model. At the same time, the participant can orient himself on the CT image displayed on a laptop computer. **b** Porcine tongue with four injected pseudotumors. The red circled areas show the pseudotumors which are slightly visible. The injection site was closed with resorbable suture material using the single-button technique. **c** CT image of the soft tissue window of a porcine tongue showing two pseudotumors as round radio-opaque masses (outlined in red) in sagittal plane. **d** Representation of two pseudotumors in ultrasound (left and right), both are outlined in red as echogenic structures. The right pseudotumor clearly shows a dorsal acoustic attenuation. **e** Macroscopic evaluation of the pseudotumor, which was cut in slices of equal thickness (3–5 mm), arranged from left to right in the order of sectioning. In the center is the white pseudotumor (outlined in red). The epithelium of the tongue mucosa (highlighted in green) is also well visible. The borders of the resection can be measured from the image. The white scale bar has a width of 20 mm. **f** Schematic representation of a pseudotumor slide. Resection borders with the corresponding categories for evaluation: positive margin (asterisk, dark purple), close margin (circle, light purple), clear margin (triangle, light green), and excessive margin (rectangle, dark green)

**Fig. 4:**
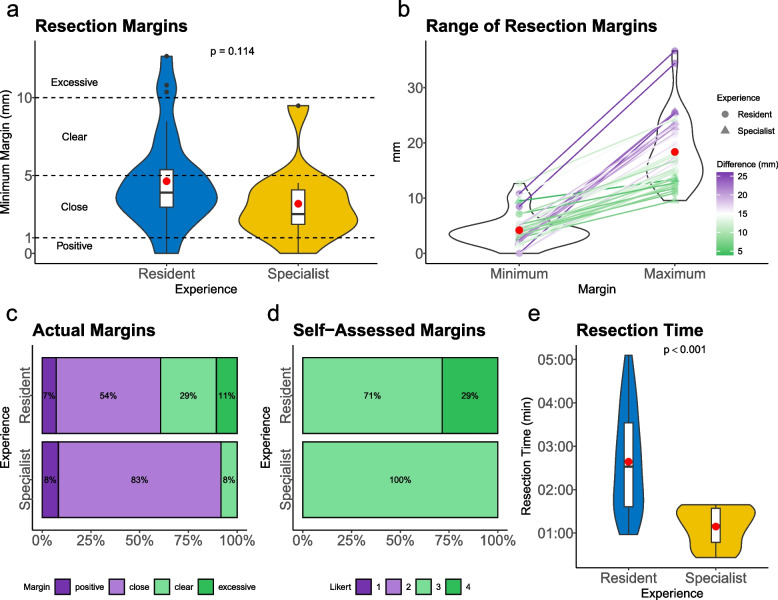
**a** Resection margins. Distribution of minimal resection margins in mm comparing residents to specialists and its *p*-value (Mann–Whitney *U* test). **b** Range of Resection Margins. Relationship between minimum and maximum resection margins in mm compare between residents and specialists. **c** Actual margins. Percentage of resection margins according to categories (positive, close, clear, and excessive margin) comparing residents to specialists. (**d**) Self-Assessed Margins. Percentage of self-assessed margins according to a 4-point Likert scale (1 = disagree to 4 = agree) comparing residents to specialists. **e** Resection-Time. Resection time in minutes comparing residents to specialists and its *p*-value (*t*-test). **a**,** b**,** e** Plots using boxplots and violin plots. Black dots are outliers, and the red dot is the mean X-axis (**a**,** b**,** e**)

In a final trial, 10 clinicians were included. An entry form was used to record the age, gender, number of biopsies on the tongue, number of resections of tongue cancer, work experience in years, and completion of residency in oral and maxillofacial surgery or oral surgery of the participants were recorded. After that each participant was asked to resect four pseudotumors using one porcine tongue, resulting in 40 resected pseudotumors from 10 porcine tongues (Fig. 3a-b). For this purpose, tongues were randomized and distributed among participants using allocated randomization rule for both settings. Available tools included a skin marking pen with a ruler (McKesson Europe AG, Stuttgart, Germany), blade scalpel (no. 11), surgical tweezers, and surgical scissors and a surgical headlight. In addition, the CT image of each tongue was visualized using a 3D slicer on a laptop computer (Fig. 3a). After the resections, each subject completed a questionnaire to evaluate the haptics of the pseudotumor, the realism of the general comparability with a tongue tumor, radiological imaging of the pseudotumors (only in the final trial), and to what extent the skill training program could be integrated into the clinical practice routine. In addition, the participants’ self-assessment of the resections performed (Fig. 4d) and the acceptance of the training model by the trial participants were surveyed by a final questionnaire (Table 2).

### Evaluation of the resection margins

Afterward, the resected specimens were fixed in formalin 4% neutral buffered (CAS#: 50–00-0) for at least 24 hours and cut into 3-mm-thick slices in the coronal plane [30]. The resection slices were documented as images with a camera (Canon EOS 80D(W), Canon Inc., Ōta, Tokyo, Japan) and a macro lens (Canon Zoom Lens EF 24–70 mm) on a reproduction stand. For each image a dissected pseudotumor, the scale of the image was first calibrated, and the smallest distance from the tumor to the resection margin was measured with a ruler using ImageJ software (version 1.54b, 14 Feb 2023 https://imagej.nih.gov/ij/download.html). We used the most common definition in the literature is as follows: positive margin (< 1 mm), close margin (1–5 mm), and clear margin (> 5 mm) [14, 15]. We added an additional category of excessive margin (> 10 mm) to have a measure of when minimal resection margins were obtained using a non-tissue-sparing technique.

### Statistical analysis

The statistical analysis was performed using the programming language R (version 4.2.2, www.r-project.org). A *p*-value < 0.05 was considered significant. The ggplot2 library was used to create the plots. Normal distribution was tested using the Shapiro–Wilk test. An unpaired *t*-test was used for normally distributed data, and a Mann–Whitney *U* test was used for non-normally distributed data.

## Results

### Participants’ characteristics

The mean age of the participants in the final trial was 35.2 years. Of the 10 participants, three were specialists. The mean work experience was 6.5 years (range: 0.5–27 years). Two of the subjects reported having performed more than 100 explicit biopsies and 50 explicit tongue tumor resections (Table 1).

**Table 1:** Participants

		Participants (*n* = 10)
**Age**	mean (SD)	35.2 (8.0)
	range	28–54
**Gender**	female	0 (0.0%)
	male	10 (100.0%)
**Specialist**	no	7 (70.0%)
	yes	3 (30.0%)
**Experience (Years)**	mean (SD)	6.5 (8.3)
	range	0.5–27.0
**Biopsies of the Tongue (Performed)**	none	1 (10.0%)
	1–10	2 (20.0%)
	> 10	3 (30.0%)
	> 50	2 (20.0%)
	> 100	1 (10.0%)
	> 1000	1 (10.0%)
**Resections of the Tongue (Performed)**	none	5 (50.0%)
	1–10	3 (30.0%)
	> 50	0 (0.0%)
	> 100	2 (20.0%)
	> 1000	0 (0.0%)

### Subjective model evaluation

The haptic palpation was rated 3.0 ± 0.5. The participants rated their ability to clearly palpate the porcine tongue to determine how many pseudotumors there were and where they were 3.5 ± 0.5. Participants were more likely to be able to clearly palpate the edge of the pseudotumor (3.2 ± 0.5) (Table 2). Consistent with this, 95.5% of the tumors in 5 test tongues (with pseudotumors varying in number from 0 to 4) could be palpated by the 10 participants during the pretrial. In one case, a nonexistent additional pseudotumor was palpated.

**Table 2:** Likert Questionnaire

Likert Questionnaire (1 = Disagree, 4 = Agree) – Mean (SD)	Final trial (*n* = 10)
I resected the pseudotumors with an adequate safety margin (10 mm).	3.2 (0.4)
I was able to be precise in this pseudotumor resection.	2.9 (0.3)
I found the time required to practice compatible with the clinical routine.	3.6 (0.5)
By practicing tumor resection using the pseudotumor model, I feel better prepared for tumor resection in a patient.	3.6 (0.5)
I can imagine the pseudotumor model being used in surgical teaching.	3.7 (0.5)
I was able to focus well during pseudotumor resection.	3.8 (0.4)
The scissors were suitable for pseudotumor resection.	3.3 (0.9)
The scalpel was suitable for pseudotumor resection.	3.6 (0.7)
I could clearly palpate where and how many pseudotumors were in a tongue.	3.5 (0.5)
The feel of the pseudotumor was comparable to a real tumor of the tongue.	3.0 (0.5)
I could clearly palpate the pseudotumor margin.	3.2 (0.6)
Based on the CT images, I was able to perform a more precise resection of the pseudotumors.	3.0 (0.9)
The radiological imaging helped me with spatial orientation.	3.3 (0.8)
I could clearly identify the pseudotumors on the CT images.	3.9 (0.3)

Regarding the imaging of the pseudotumor model, participants reported being able to clearly identify the pseudotumors on CT images (3.9 ± 0.3). Therefore, the participants rated radiological imaging as helpful in spatial orientation (3.3 ± 0.8). In addition, the majority (3.0 ± 0.9) agreed with the statement that they could perform a more precise resection of the pseudotumors based on the CT scan. Overall, the participants could envision the skill training program becoming established in surgical education (3.7 ± 0.5) (Table 2).

### Resection duration

The mean duration of tumor resection was 2:12 ± 1:13 (min:s [mean ± sd]), making training rapidly feasible. The mean duration of the residents was 2:39 ± 1:10, and significantly slower (*t*-test, *p* < 0.001) to the specialists with a mean duration of 1:09 ± 0:27 (Fig. 4e). The time spent practicing with the pseudotumor model was rated as compatible with clinical routines (3.6 ± 0.5). The statement “by practicing tumor resection using the pseudotumor model, I feel better prepared for tumor resection in a patient” had an overall positive response of 3.6 ± 0.5 (Table 2).

### Resection margins

The mean minimum resection margin of the pseudotumors was 4.2 ± 1.2 mm (mean ± SD; median 3.8 mm), whereas the mean minimum resection of residents was 4.6 ± 3.1 mm (mean ± SD; median 3.9 mm) and that of specialists was 3.2 ± 2.4 mm (mean ± SD; median 2.5 mm) (Fig. 4a), which did not differ significantly (Mann–Whitney *U* test, *p* = 0.114). At the same time, the maximum resection margin measured reached 18.4 ± 6.1 mm (median: 17.2 mm) (Fig. 4b). In terms of resection margins, our results showed 40 pseudotumors with at least 3 positive margins (7.5%), 25 resections with close margins (62.5%), 9 tumors with clear margins (22.5%), and 3 excessive margins (7.5%). There were 28 pseudotumors resected by residents, with 7.1% positive margins, 53.6% resection with close margins, 28.6% clear margins and 10.7% excessive margin. The specialists, on the other hand, performed 12 resections. This resulted in 8.3% positive margins, 83.3% close margins, and 8.3% clear margins and 0% excessive margin (Fig. 4c). In contrast, participants tended to agree (3.2 ± 0.4; 4-point Likert scale; 1 = disagree to 4 = agree) that they resected the pseudotumors with an adequate safety margin (10 mm) (Fig. 4d). Their own ability to resect the pseudotumors accurately was rated as 2.9 ± 0.3. During resection, the participants were able to focus well (3.8 ± 0.4). The scalpel (3.6 ± 0.7) was the preferred instrument for resection compared to scissors (3.3 ± 0.9) (Table 2).

## Discussion

In this study, we developed and validated a pseudotumor model for skill training in biopsy and the resection of OC using fresh cadaveric porcine tongues that met the pre-specified requirements. To the best of our knowledge, this is the first tongue pseudotumor model for off-patient biopsy and resection training with feedback on performance.

In the past, surgical training often involved the use of live pigs [31], perfused porcine tissue [32], or porcine cadavers [33]. For example, Teoh et al. created a bladder tumor using porcine cadavers by impaction of the bladder wall [34], while Chauvet et al. used a pseudotumor model to mimic renal tumors to allow the performance of laparoscopic tumor resection [35]. A curriculum focused on teaching robotic resection of oropharyngeal cancer showed that use of the training program with a porcine oropharynx model improved resection peripheral margins from 3.3 ± 1.8 mm (mean ± sd) pre-curriculum to 5.2 ± 0.7 mm post-curriculum and for deep 2.9 ± 2.5 mm to 5.1 ± 1.11 mm respectively, after an average total training time of 388 ± 57 minutes [23]. Another study (RCT) showed a reduction in positive margins (control: from 60 to 30%; real-time visual computer navigation feedback: from 80 to 0%) using a training program on self-made plastic models, but did not provide information on margins in mm [22]. Interestingly, a study of robotic radical prostatectomy showed that surgical experience correlated with improved site-specific surgical margins [36]. In this regard, human factor remains a major challenge and efforts should be made to reduce the potential negative effects of inadequate biopsy and tumor resection. Promising in this regard is the fact that distance estimation [37] and visual-spatial ability [38] are trainable. These training programs could improve overall resection margins, thereby positively impacting patient outcomes. For example, a training program in rectal cancer using an adapted surgical technique (total mesorectal excision) for better margin control led to a significant improvement in survival [39].

However, these results are not directly applicable to OC, as robotic resections are not state-of-the-art for OC. Furthermore, one model was equipped with a foam layer which is supposed to represent an endophytic tumor [23]. However, the main concern is that it does not correspond to real tumors that spread in depth [18], while the biopsy is limited to the immediate surface, which does not allow training of spatial resection. Finally, it lacks the typical haptic characteristics of an OC.

In contrast, our proposed pseudotumor model more resembles the spatial expansion of OC. The use of porcine tongues has a clear advantage over the limited availability of patients or human cadaveric tongues and also provides more room for training due to their increased length (in our sample, 15.8 cm × 5.1 cm [mean maximum length x width]) compared to human tongues (9.0 × 6.4 cm) [40]. The use of porcine tongues from butchery as food is safe, as they must meet the relevant legal safety requirements [27, 28]. In contrast, the use of 3D printed models still does not provide the same level of realism as soft cadaveric tissue and would not be considered equivalent [41]. Furthermore, both the porcine tongues and the materials used are inexpensive, allowing frequent use to during a training program and can be stored by cryopreservation (Fig. 2d). Moreover, our developed model could be further improved by preserving it with a chemical solution [42].

Within the developed model the pseudotumors appear radiologically strong hyperdense and homogeneous, which clearly facilitates the training process (Fig. 3c-d). In addition, beam hardening artifacts often render CT images of the oral and maxillofacial region difficult to evaluate. The model, on the other hand, offers optimal radiological training conditions and focuses on acquiring accurate tumor resection and biopsy skills. The presented pseudotumor model can be imaged with US (Fig. 3d) and is therefore also suitable for US-guided margin resection training.

Overall, our study confirmed the need for training in the margin control. 70% of resections had an inadequate margin (≤ 5 mm), which is comparable to the aforementioned margins in real oncological surgery [15, 17, 19]. Interestingly, in OC resection, initial intraoperative margins (< 5 mm vs. ≥ 5 mm) already have a negative impact on the rates of local (33.8% vs. 17.2%) and distant (13.7% vs. 0%) recurrence [21]. Consistent with this, a meta-analysis found that revision from positive margins (< 1 mm) to non-positive margins (≥ 1 mm) with frozen resection guidance resulted in worse survival than initially negative margins (≥ 1 mm) (HR 2.592, 95% CI 1.873–3.588) [20]. As a result, the patient’s outcome due to a poor initial resection margin could not be corrected by additional resections [20, 21].

To make matters worse, even for specialist in oral and maxillofacial surgeons, the situation is not better, because despite the given requirement of a resection distance of at least 10 mm [16], close margins (< 5 mm) have been reported to vary between 11 and 45%, and positive margins between 7 and 43% respectively [15, 17, 19]. Yet, resection margins are critical in determining outcomes, including survival and recurrence rates [14]. Patients with clear margins (> 5 mm) were found to have a higher disease-free survival rate than those with close or positive margins (< 1 mm) (5-year probability, 0.78 vs. 0.43 and 0.29, respectively) and a lower hazard ratio for recurrence (0.22, 95% CI 0.07–0.71) [19]. A meta-analysis showed that a clear margin resulted in an absolute risk reduction of 24% for recurrence (95% CI 12–30%) [17]. Furthermore, inadequate resection margins contributed to increased morbidity rates and costs [14].

At the same time, in our study, participants tended to agree (3.2 ± 0.4; 4-point Likert scale; 1 = disagree to 4 = agree) that they resected the pseudotumors with an adequate safety margin of 10 mm. This discrepancy between self-assessment and actual performance magnifies the problem of numerous inadequate tumor resections. In this context, the substantial underperformance cannot be explained by tissue shrinkage under tension during surgery (21.2–25.6%) [43] or formalin fixation (10%) [14].

When evaluating the margins, we observed that the resection distance was often asymmetric (Fig. 3e and Fig. 4b). One explanation for this could be that the spatial position of the tumor within the resection specimen was incorrectly proposed by the participants. This is astonishing because in our case, the conditions were ideal; there was no bleeding, no constriction in the oral cavity, and definite borders of the pseudotumor. This is consistent with the reported resection margins, which vary widely from center to center [15, 17, 19], or the fact that the mean human error in specimen repositioning at 5 minutes was 9 mm at the mucosal margins and 12 mm in the deep tumor bed [44].

In the past, efforts have been made to address this issue by improving tumor resection through the use of technology, such as fluorescent marking of tumors to identify narrow or positive margins [45, 46], an ultrasound (US)-based margin control [47, 48], or in the application of robotics [23, 49]. However, the aforementioned approaches are either not used in practice or do not eliminate human error regarding the initial resection margin, which determines the patient’s outcome. The fluorescent marker shows only directly exposed tumor tissue as a fluorescent spot, which is helpful to avoid leaving tumor tissue in place [46]. The US approach actually improves the minimum resection margin from 3.5 ± 2.0 mm to 4.9 ± 2.5 mm, but is still far from a solid clear margin [48]. The robotic approaches are still in the experimental stage and only guide the superficial resection margins around the visible portion of the tumor [49]. However, the proposed model could be combined with all three approaches (fluorescence, US, and robot-assisted) or used to train each of these approaches outside the patient.

Dentists in particular have an outstanding role in the early detection of OC [50]. No other specialist performs intraoral inspection with the regularity. In this regard, biopsy is the gold standard for the diagnosis of OC and therefore must be routinely mastered especially by dentists [51]. The current standard for training biopsies and tumor resections is on the patient under the supervision of an experienced dentist or surgeon. However, this has several limitations. Patients must be available, which is not always the case. Since mistakes during biopsy and tumor resection cannot be corrected [12, 20, 21], the barrier to teaching students or younger colleagues is very high. Yet, a training program using the presented pseudotumor model could be established as standard in dentistry and would allow dental students, dentists and residents in oral and maxillofacial surgery to repeat the performance of tumor palpation, biopsies, and resection until they are confident in achieving an adequate biopsy technique and resection margin in real patients. A direct performance feedback is possible (Fig. 3e-f) so that a learning curve could be tracked. The skill training using the pseudotumor model could therefore reduce the risk for the occurrence of insufficient safety distance on real patients and thus decisively improve the prognosis and probability of survival. Just as practice models for extractions or microvascular suturing techniques are an integral part of dental and medical education, practice of biopsies and resections could become a regular part of the curriculum. The pseudotumor model can be produced inexpensively in any location and stored in a freezer. If slaughterhouse discards are used, the model is very cost-effective. With a mean resection time of 2:12 ± 1:13 (min:s [mean ± sd]), a sufficient number of training sessions could be incorporated. Depending on the local culture, other species (sheep, goat, cow, etc) could be considered.

Nevertheless, the following limitations should be considered. We did not implement a long-term training in our study, as this was clearly beyond the scope. This study focused on the development and validation of the model. It can be used to assess the suitability of the model for practice biopsies and resections and its acceptability. Nevertheless, the results of this study can be used for sample size calculations in confirmatory studies investigating the training effect using the presented model.

## Conclusion

Although, participants regardless of training level tended to agree that they had resected the pseudotumor with an ideal safety margin, both the observed and reported resection minimum margins were inadequate. This highlights the need for the implementation of a skill training for OC surgery. Our study demonstrated that the presented pseudotumor model is suitable as a realistic training tool, is inexpensive and can be produced in any number. It can be stored by cryopreservation and provides a safe environment for the training and immediate evaluation of tumor resection performance. The skill training with the introduced pseudotumor model could be incorporated into dental, medical, or oncological surgery curricula. Future studies should evaluate the long-term training effects using this model and its potential impact on improving patient outcomes.

## Data Availability

The datasets used and/or analysed during the current study are available from the corresponding author on reasonable request.

## Abbreviations

CT: Computed tomography
HU: Hounsfield unit
US: Ultrasound
wt/vol%: Weight/volume%
